# Bouveret’s syndrome: presentation of two cases with review of the literature and development of a surgical treatment strategy

**DOI:** 10.1186/1471-2482-13-33

**Published:** 2013-09-04

**Authors:** Felix Nickel, Matthias M Müller-Eschner, Jackson Chu, Hendrik von Tengg-Kobligk, Beat P Müller-Stich

**Affiliations:** 1 Department of General, Visceral and Transplantation Surgery, University of Heidelberg, Im Neuenheimer Feld 110, 69120, Heidelberg, Germany; 2 Department of Diagnostic and Interventional Radiology, University of Heidelberg, Heidelberg, Germany; 3 Department of Radiology, German Cancer Research Center (dkfz), Heidelberg, Germany; 4 Institute of Diagnostic, Interventional and Pediatric Radiology, University Hospital Bern, Inselspital, Bern, Switzerland

## Abstract

**Background:**

Bouveret’s syndrome causes gastric outlet obstruction when a gallstone is impacted in the duodenum or stomach via a bilioenteric fistula. It is a rare condition that causes significant morbidity and mortality and often occurs in the elderly with significant comorbidities. Individual diagnostic and treatment strategies are required for optimal management and outcome. The purpose of this paper is to develop a surgical strategy for optimized individual treatment of Bouveret’s syndrome based on the available literature and motivated by our own experience.

**Case presentation:**

Two cases of Bouveret’s syndrome are presented with individual management and restrictive surgical approaches tailored to the condition of the patients and intraoperative findings.

**Conclusions:**

Improved diagnostics and restrictive individual surgical approaches have shown to lower the mortality rates of Bouveret’s syndrome. For optimized outcome of the individual patient: The medical and perioperative management and time of surgery are tailored to the condition of the patient. CT-scan is most often required to secure the diagnosis. The surgical approach includes enterolithotomy alone or in combination with simultaneous or subsequent cholecystectomy and fistula repair. Lower overall morbidity and mortality are in favor of restrictive surgical approaches. The surgical strategy is adapted to the intraoperative findings and to the risk for secondary complications vs. the age and comorbidities of the patient.

## Background

Bouveret’s syndrome occurs when a gallstone enters the small bowel via a bilioenteric fistula and is impacted in the duodenum or stomach, causing gastric outlet obstruction. It is a rare form of gallstone ileus that results from gallstone disease and causes significant morbidity and mortality rates of up to 30% [1]. Bouveret’s syndrome is more prevalent in the elderly and in females, with a reported median age of 74 years and a female to male ratio of 1.9 [1-4]. The formation of a bilioenteric fistula is initiated when the walls of the biliary system and the bowels are chronically inflamed and adherent. Increasing intraluminal pressure caused by obstruction leads to local ischemia and necrosis, allowing the gallstone to perforate the walls and pass into the bowels [5]. Of all patients with gallstones, between 0.3 and up to 5% are reported to develop bilioenteric fistulas [6,7]. Stones smaller than 2.5 cm usually pass into the small bowel where they can pass uneventfully or cause gallstone ileus which is more frequent with larger stones [8]. The majority of gallstones impact distally in the terminal ileum and obstructions in the duodenum are less common [9]. Two cases of Bouveret’s syndrome are presented with individual management and restrictive surgical approaches tailored to the condition of the patients and intraoperative findings. The purpose of this paper is to develop a surgical strategy for optimized individual patient treatment for suspected Bouveret’s syndrome based on the available literature and motivated by our own experience.

## Case presentation

### Case 1

A 78 year old Caucasian male presented to the Emergency Department with a six week history of upper abdominal pain, discomfort, nausea, subsequent hematemesis with coffee-ground material, and a four day history of diarrhea and vomiting with diffuse abdominal pain. He had not sought prior medical help for the symptoms of this episode. He was found alert but disoriented at presentation. Past medical history of the patient included type 2 diabetes mellitus, arterial hypertension with subsequent hypertensive crisis, hyperlipidemia and cardiac conditions comprised of coronary heart disease, atrial fibrillation with absolute arrhythmia, left bundle branch block and poor left ventricular function. At time of presentation, the patient was hypotonic with a systolic blood pressure of 70 mm Hg and tachyarrhythmic at 120–150 beats per minute. Initial laboratory investigations revealed severe electrolyte imbalance and volume depletion with hypokalemia (2.1 mmol/L), hyponatremia (119 mmol/L), a hemoglobin content of 15.9 g/dL and signs of inflammation with a leukocyte count of 35/ nL and c-reactive protein of 55.7 mg/L. The patient was started on vasopressor therapy with volume and electrolyte resuscitation, and was admitted to the intensive care unit for hemodynamic monitoring. A nasogastric tube was placed and drained 3 L of gastric contents. Intravenous antibiotics and proton pump inhibitor therapy were started. Due to respiratory failure, the patient was intubated and was mechanically ventilated.

**Figure 1:**
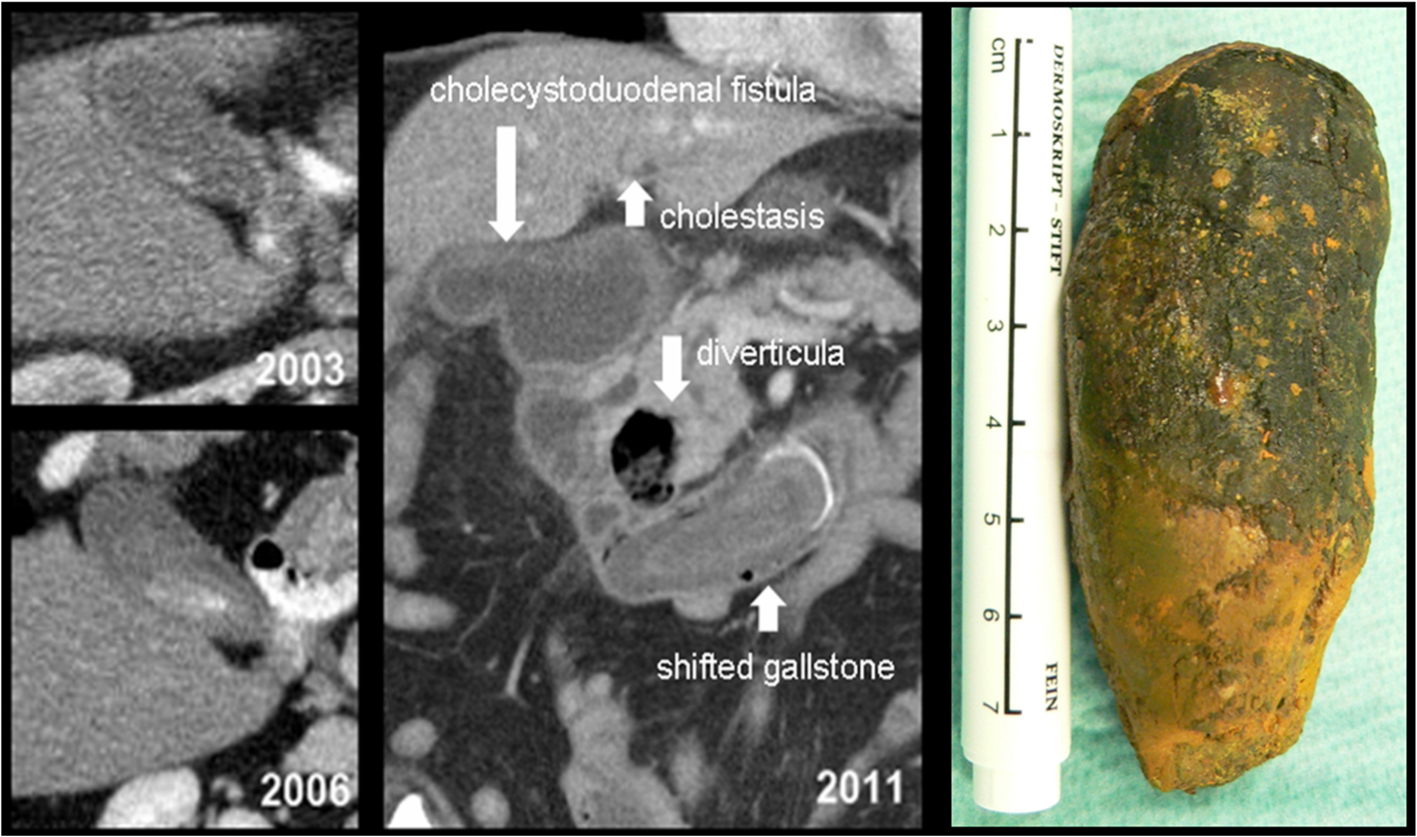
**Case 1, axial slices of CT scans performed in 2003 and 2006 showing calculus development over time.** Double-oblique MPR of the CT scan performed in 2011 at hospitalization, with delineation of the cholecystoduodenal fistula, the duodenal diverticula and the shifted gallstone. Retrieved gall stone on the right.

A thorough review of the patient’s past medical records revealed a history of a duodenal ulcer and duodenal diverticula: 8 years prior to the current episode the patient had presented with mild cholangitis that was successfully treated with i.v. antibiotics. At that time CT was performed due to an elevated carcinoembryogenic antigen to rule out malignant disease. CT scan showed duodenal diverticula as well as a gallbladder filled with sludge but no delineation of concretions or malignancy (Figure 1). 5 years prior to the current episode the patient had already presented with an episode of abdominal distention and constipation and a CT scan from this episode showed duodenal diverticula, a sludge-filled gallbladder with a calculus of 3 cm and multiple smaller concretions up to 0.5 cm in size but without signs of cholestasis or cholecystitis.

Imaging by plain x-rays was suspect for free air in the abdomen. Esophagogastroduodenoscopy was performed, showing a normal esophagus and a dilated stomach with excessive secretions and mixed gastric contents. In the proximal duodenum, a cholecystoduodenal fistula was delineated. In the duodenal C-loop adjacent to the papilla a 1 cm diverticulum was present, and a concrement was found occluding the lumen of the distal duodenum. The concrement could not be mobilized endoscopically. Abdominal CT scan showed a contained rupture of the gallbladder with a large fistula to the duodenal c-loop (Figure 1). A 7.6 cm partially calcified gall stone was located in the distal duodenum (Figure 1). Intrahepatic and extrahepatic cholestasis was found caused by compression of the distal DHC by the gallstone and a duodenal diverticulum. CT scan showed three more diverticula in the descending part of the duodenum.

The initial management strategy for this elderly patient in septic condition with significant cardiac comorbidities and hemodynamic instability was to continue conservative treatment in the intensive care unit. On the fourth day after admission, the patient’s respiratory condition, inflammatory state, and hemodynamic situation had improved. He was still tachycardic at 120 beats per minute but stable with low doses of vasopressors. Laparotomy was performed to remove the stone since endoscopy had failed. A 3 cm longitudinal jejunostomy was made at a 10 cm distance to the Ligament of Treitz and the gallstone retrieved (Figure 1). An area of pressure necrosis on the duodenal wall at the impacted site was subsequently repaired with a running suture. The jejunal incision was closed with a transverse suture in the fashion of a stricturoplasty to avoid development of stenosis. Given the patient’s comorbidities and complicated hemodynamic situation, along with the strategy to minimize the operative trauma and procedural time, the cholecystoduodenal fistula was left in place and covered by an omentum patch. In the postoperative course the patient’s pulmonary and hemodynamic situation improved and stabilized quickly. On the third postoperative day the inflammatory parameters had improved and antibiotic treatment was discontinued. After an uneventful further postoperative course the patient was transferred to a rehabilitation centre.

### Case 2

**Figure 2:**
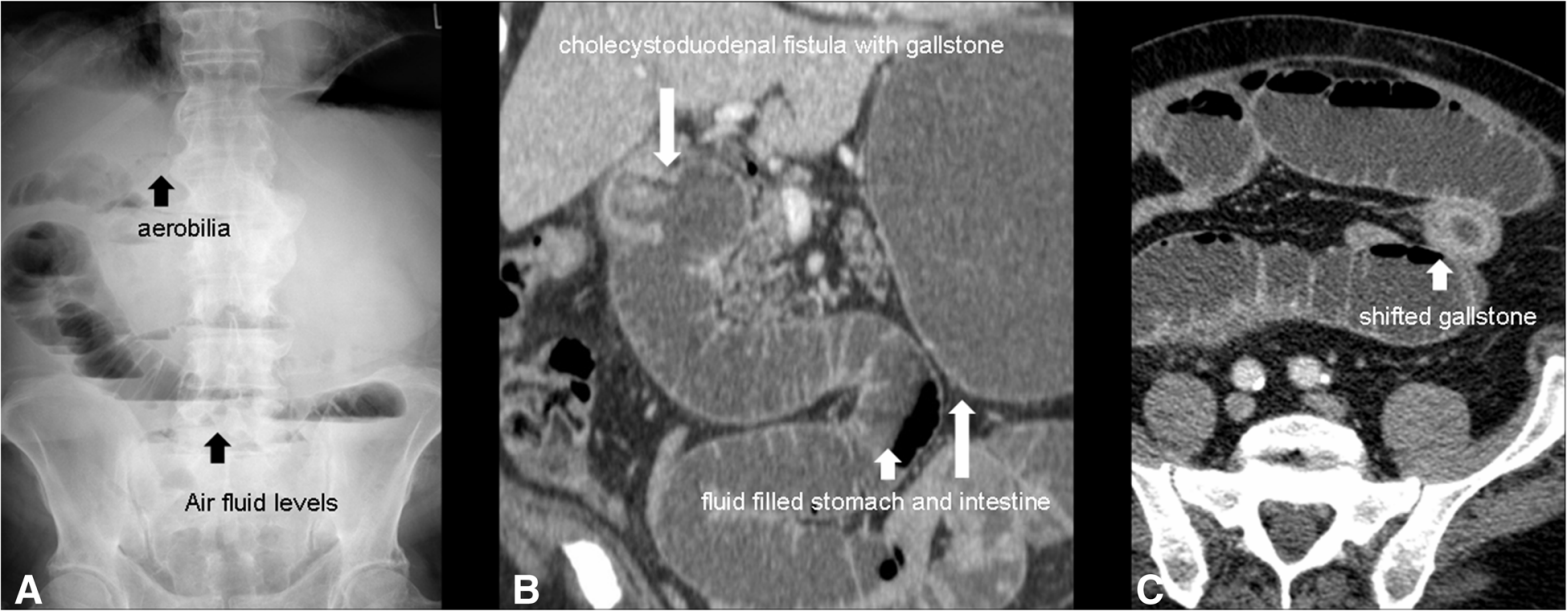
Case 2, A: X-ray with multiple air fluid levels as a sign of ileus, B: double-oblique MPR of the CT scan showing the gallstone within a cholecystoduodenal fistula and a fluid filled stomach and proximal intestine C: axial slice of the CT scan with delineation of the shifted gallstone in the jejunum.

A 75 year old Caucasian male was admitted with tachyarrhythmia, atrial fibrillation, and hypertension escalating in a hypertensive crisis. His past medical history was significant for multiple cardiovascular pathologies including atrial fibrillation with ablation treatment, coronary artery disease with a stent placed in the left anterior descending coronary artery, arterial hypertension with subsequent hypertensive crisis, and mild insufficiency of the aortic, mitral, and tricuspid valves. Four days after admission, he developed mechanical ileus and abdominal CT scan was obtained after abdominal X-ray revealed pneumobilia and signs of ileus (Figure 2). CT revealed a fluid-filled stomach, duodenum and proximal jejunum as a sign of mechanical ileus. The gallbladder base showed inflammatory changes with fluid retention, air inclusions and a gallstone within a cholecystoduodenal fistula. Another gallstone was found within a jejunal loop in the left lower abdomen and consecutively collapsed bowel loops distal to the stone. CT scan also revealed a 4.4 cm duodenal diverticulum adjacent to the papilla (Figure 2).

Laparotomy revealed a dilated segment of proximal jejunum that was obstructed by a 4 cm gallstone 100 cm distal to the Ligament of Treitz. Another gallstone of a similar size of 4 cm was found in a cholecystoduodenal fistula with an abscess that was covered by omentum. Because of the poor general condition of the patient and his significant comorbidities and hemodynamic instability, the risk for performance of Whipple’s procedure was considered substantial. The restrictive surgical approach consisted of pericholecystitic abscess drainage and cholecystectomy, gallstone extraction by jejunostomy, resection of a short segment of jejunum around the jejunostomy site and jejunoduodenostomy was performed to repair the defect in the duodenal wall with Roux en Y jejunojejunostomy. Post-operatively, the patient was still tachycardic, remained intubated and ventilated, and was dependent on high dose vasopressors. Two thrombi were discovered in the left atrium by ultrasonography; thus, he was started on full dose heparin and medically converted to sinus rhythm following thrombolysis. He did well after a course of antibiotic therapy and was discharged in improved general condition.

### Discussion and literature review

The diagnosis of Bouveret’s syndrome is best made as early as possible, as the risks of morbidity and mortality are high given the affected patient population with frequent cardiovascular and general comorbidity [9,10]. The clinical signs and symptoms of Bouveret’s syndrome are variable and nonspecific. The most common symptoms are described as a triad consisting of epigastric pain, nausea, and vomiting [11,12]. Other common non-specific signs are abdominal tenderness, dehydration, abdominal distention, upper gastrointestinal hemorrhage with hematemesis, and pyrexia [1,13]. Differential diagnoses include perforated peptic ulcer, pancreatitis, gastric volvulus, bezoars and malignant fistula [14-16]. It is important to consider the diagnosis of Bouveret’s syndrome in patients being over the age of 70 years or having a past history of symptomatic gallstone disease [15,17]. Interestingly, the presence of duodenal diverticula, as seen in both presented cases, has been previously studied for an association with gallstone complications. An analysis of 350 patients who received ERCP and were diagnosed with juxtapapillary duodenal diverticula (JPDD) reported that those affected had a higher incidence of cholecystolithiasis (29.4% vs. 20.8%; p = 0.039), and choledocholithiasis (46% vs. 33.1%; p<0.001) and their recurring episodes (6.6% vs. 1.4%; p = 0.002) compared to a matched pair control group without JPDD [18]. The conclusions of other studies state that JPDD are linked with choledocholithiasis but cholecystolithiasis was only linked to larger JPDD, suggesting that size may be associated with an increased risk [19-22]. The pathophysiology of JPDD leading to gallstones is not exactly known but the currently available data suggests that JPDD may increase the risk of gallstone disease and therefore can be a risk factor for Bouveret’s syndrome.

### Diagnostics

The use of imaging studies in combination with the clinical presentation is important for recognizing Bouveret’s syndrome [23]. Imaging of the abdomen by plain x-rays is the appropriate initial step but is alone diagnostic of Bouveret’s syndrome in only 21% of cases [24]. Rigler’s triad, described as small bowel obstruction, pneumobilia, and an ectopic gallstone, is specific for gallstone ileus but is not readily seen on plain films nor complete in most patients [25]. Ultrasound of the abdomen is recommended to evaluate for possible cholecystitis and can also reveal a dilated stomach [26-28]. It has been used for the diagnosis of Bouveret’s syndrome based on the demonstration of pneumobilia and the ectopic location of the gallstone [29-31]. Disadvantages of ultrasound include difficulties locating the gallstone and being ineffective when there is excessive intestinal gas [32,33]. In the majority of cases (60%) CT scan is used for diagnosis of Bouveret’s syndrome [7,34-37]. A CT scan can provide important information about the presence of a fistula, the inflammatory state of the surrounding lumen and tissue, and the size, number and locations of the occluding gallstones. CT more commonly identifies Rigler’s triad than plain x-rays and contrast agent helps identify abscess formation [7,11,36,37]. However, 15 to 25% of gallstones are isoattenuating and therefore not well visualized on CT scan. In those cases, magnetic resonance cholangiopancreatography (MRCP) can be used additionally to help find the diagnosis and to rule out the presence of intraductal concrements. However, difficulties in interpretation may occur as concrements and air are difficult to be differentiated by MRCP. MR Imaging is also sensitive for the presence of a fistula and can be used for confirmation of findings before treatment [2,11,37]. In 69% of the cases the impacted stone can be visualized in Esophagogastroduodenoscopy and Bouveret’s syndrome can be affirmed. A dilated stomach with food content, duodenal ulcer with excessive inflammation and edema, and cholecystoduodenal fistula may also be seen by endoscopy. A first attempt to remove the stone can be done simultaneously during endoscopy, however this only successful in the minority of cases. In about 20% to 40% of all cases the final diagnosis will only be established during surgery [16].

### Treatment

Many authors have adopted the stance that endoscopic or percutaneous approaches should always be attempted prior to surgery [2,38-40]. The main reason for this is that Bouveret’s syndrome tends to affect the elderly, who will likely be poor surgical candidates in the presence of multiple comorbidities that can lead to operative complications [21]. Methods of endoscopic or percutaneous treatment as well as the references to successful cases are as follows: mechanical lithotripsy, laser lithotripsy, extracorporeal shockwave lithotripsy, and intracorporeal electrohydraulic lithotripsy [2,38-44]. However, despite some reports of success, 91% of patients will have to undergo surgery for definitive treatment [45-50]. The size of the gallstone is an important factor to consider because especially stones larger than 2.5 cm are difficult to remove endoscopically and may also become impacted in the esophagus [2,45-49]. This is consistent with the low success rates of endoscopic treatments since the stones that cause gastric outlet obstruction tend to be large [16,17]. If fragmentation of a large gallstone is attempted, there can be an increased risk of causing a distal gallstone ileus [51,52]. Other risks of endoscopic treatment include hemorrhaging or perforating the intestinal wall during intracorporeal electrohydraulic lithotripsy [2].

**Figure 3:**
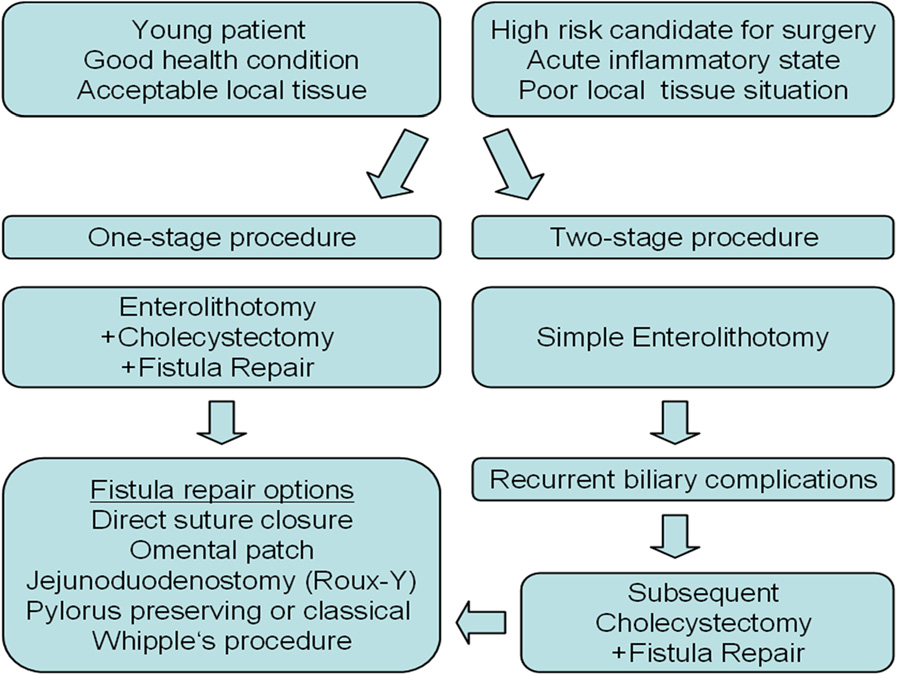
Surgical treatment strategy for Bouveret’s syndrome.

The main treatment modality for Bouveret’s syndrome is surgery, especially when percutaneous or endoscopic approaches are not an option or have failed [53]. The most common surgical options for extracting the stone are enterolithotomy and gastrotomy [16,54]. Small bowel obstruction should be managed by enterolithotomy with longitudinal antimesenteric incision. A transverse closure of the enterolithotomy site is recommended to avoid stenosis [27,28]. Resection is required for parts of the small bowel that have become irreversibly damaged [54,55]. Surgical removal of the stone can be carried out with or without a simultaneous (one stage procedure) or subsequent cholecystectomy and fistula repair (two stage procedure) [15-17,27,28]. Cholecystectomy is indicated for retained gallstones in the gallbladder to prevent recurrence and complications [10,54]. Laparoscopic techniques should be considered for surgical treatment whenever possible to minimize the physiological trauma. However, higher rates of conversion are seen in these difficult cases [56,57]. With an unrepaired bilioenteric fistula the patients are at risk for biliary disorders including cholecystitis and cholangitis. Previous studies have suggested an incidence as high as 56% [25]. However, a review of 1001 cases of gallstone ileus showed that only 10% of patients required reoperation with fistula repair due to persistent biliary symptoms [17]. An elevated risk for the development of gallbladder carcinoma has been discussed with persistent bilioenteric fistula but to date there is no evidence in favor of this argument [58,59]. Supporters of a simple stone extraction procedure claim that the fistula can close spontaneously if there are no residual stones in the gallbladder and the cystic duct is patent [1,54]. No convincing data has shown which approach provides better outcomes when applied to all cases [60-62]. We suggest a surgical treatment strategy that is adapted to the individual patient when endoscopic treatment is not an option or has failed (Figure 3). Stone extraction alone is advised in elderly patients with associated comorbidities due to the risk of operative complications and in case of acute inflammatory state due to the high rates of fistula closure breakdown with the poor local tissue conditions in the acute inflammatory state [25]. In older and comorbid patients in acceptable condition with non acute inflammatory tissue conditions at surgery a one stage procedure with fistula repair and cholecystectomy may be considered to avoid secondary complications. However, the perioperative morbidity and mortality rates are in favor of simple enterolithotomy and subsequent fistula repair can be performed when needed [16,17,54]. Younger patients with higher life expectancy seem more likely to develop secondary complications over time due to persistent bilioenteric fistula and would thus implicate simultaneous fistula repair in a one stage procedure in case of acceptable health condition and absence of acute inflammatory state. Subsequent fistula repair should be performed in the case of recurrent biliary complications with absence of spontaneous fistula closure. The mortality rate of Bouveret’s syndrome, which was previously as high as 30%, is more recently reported to be around 12% due to improved diagnostic techniques and more restrictive surgical approaches [1,54].

## Conclusion

Bouveret’s syndrome is a rare but potentially life threatening condition with gastric outlet obstruction caused by large gallstones. Treatment can be delayed due to the unspecific symptoms and extended diagnostic workup. CT scan is in most cases required for diagnosis and the patients often require intensive care treatment for hemodynamic stabilization. The underlying obstructive disease can lead to further deterioration of the patient’s condition and should be treated as soon as possible. Percutaneous and endoscopic treatments can in some cases be successful as first line treatment. Most patients have to undergo surgery for stone extraction. Simultaneous or subsequent cholecystectomy and fistula repair should be considered according to the patient’s age, general and actual health status, the local tissue conditions found during surgery, as well as occurrence or risk for secondary biliary complications. An individual treatment and management strategy with optimal timing of surgery, and a restrictive surgical approach should be considered as a basis for optimal patient outcome. The mortality rate was lowered over time from 30% to 12% due to improved diagnostics and more restrictive surgical approaches.

### Consent

Written informed consent was obtained from the patient or his next of kin for publication of the cases and accompanying images. A copy of the written consent is available for review by the Editor of this journal.

## Competing interests

The authors declared that they have no competing interests.

## Authors’ contributions

FN, MME, HTK and BPM have made substantial contributions to conception and design of the study, and interpretation of data. FN, MME, and JC have made substantial contributions to acquisition and analysis of data. FN, MME, and JC have been involved in drafting the manuscript. HTK and BPM have been involved in revising it critically. All the authors have given final approval of the version to be published.

## Pre-publication history

The pre-publication history for this paper can be accessed here:

http://www.biomedcentral.com/1471-2482/13/33/prepub
